# Supplementary material for: Differential coexistence of multiple genotypes of Ophiocordyceps sinensis in the stromata, ascocarps and ascospores of natural Cordyceps sinensis
Source: PLoS One. 2023 Mar 9;18(3):e0270776. doi: 10.1371/journal.pone.0270776 (PMC9997936; doi:10.1371/journal.pone.0270776)
Supplement: S1 File — (ZIP) [file pone.0270776.s001.zip › data file for Figure 2 transition mutants.pptx]

## Slide 1
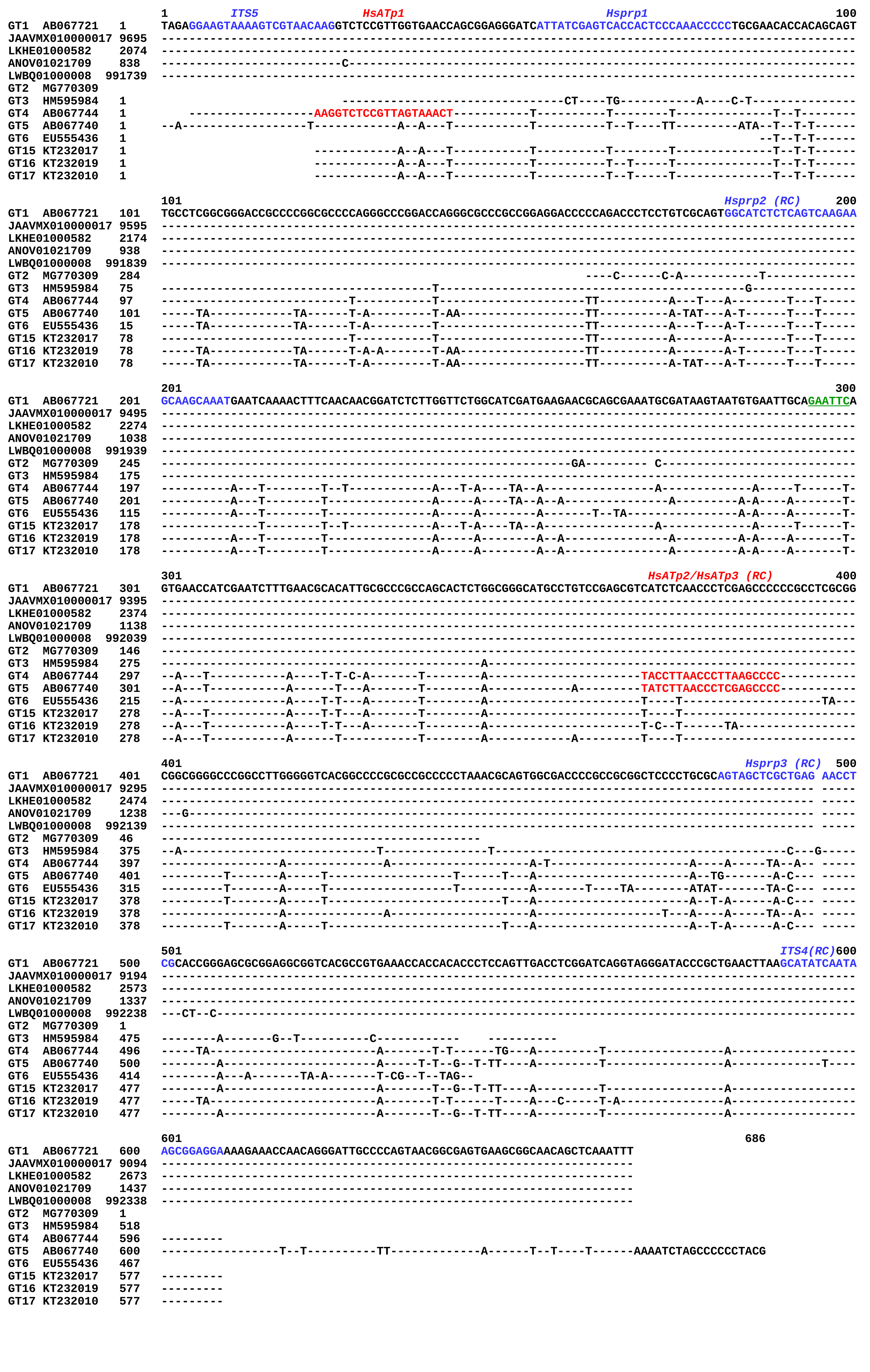

1 ITS5 HsATp1 Hsprp1 100
GT1 AB067721 1 TAGAGGAAGTAAAAGTCGTAACAAGGTCTCCGTTGGTGAACCAGCGGAGGGATCATTATCGAGTCACCACTCCCAAACCCCCTGCGAACACCACAGCAGT
JAAVMX010000017 9695 ----------------------------------------------------------------------------------------------------
LKHE01000582 2074 ----------------------------------------------------------------------------------------------------
ANOV01021709 838 --------------------------C-------------------------------------------------------------------------
LWBQ01000008 991739 ----------------------------------------------------------------------------------------------------
GT2 MG770309
GT3 HM595984 1 --------------------------------CT----TG-----------A----C-T---------------
GT4 AB067744 1 ------------------AAGGTCTCCGTTAGTAAACT-----------T----------T--------T--------------T--T--------
GT5 AB067740 1 --A------------------T------------A--A---T-----------T----------T--T----TT---------ATA--T--T-T------
GT6 EU555436 1 --T--T-T------
GT15 KT232017 1 ------------A--A---T-----------T----------T--------T--------------T--T-T------
GT16 KT232019 1 ------------A--A---T-----------T----------T--T-----T--------------T--T-T------
GT17 KT232010 1 ------------A--A---T-----------T----------T--T-----T--------------T--T-T------
 101 Hsprp2 (RC) 200
GT1 AB067721 101 TGCCTCGGCGGGACCGCCCCGGCGCCCCAGGGCCCGGACCAGGGCGCCCGCCGGAGGACCCCCAGACCCTCCTGTCGCAGTGGCATCTCTCAGTCAAGAA
JAAVMX010000017 9595 ----------------------------------------------------------------------------------------------------
LKHE01000582 2174 ----------------------------------------------------------------------------------------------------
ANOV01021709 938 ----------------------------------------------------------------------------------------------------
LWBQ01000008 991839 ----------------------------------------------------------------------------------------------------
GT2 MG770309 284 ----C------C-A-----------T-------------
GT3 HM595984 75 ---------------------------------------T--------------------------------------------G---------------
GT4 AB067744 97 ---------------------------T-----------T---------------------TT----------A---T---A--------T---T-----
GT5 AB067740 101 -----TA------------TA------T-A---------T-AA------------------TT----------A-TAT---A-T------T---T-----
GT6 EU555436 15 -----TA------------TA------T-A---------T---------------------TT----------A---T---A-T------T---T-----
GT15 KT232017 78 ---------------------------T-----------T---------------------TT----------A-------A--------T---T-----
GT16 KT232019 78 -----TA------------TA------T-A-A-------T-AA------------------TT----------A-------A-T------T---T-----
GT17 KT232010 78 -----TA------------TA------T-A---------T-AA------------------TT----------A-TAT---A-T------T---T-----
 201 300
GT1 AB067721 201 GCAAGCAAATGAATCAAAACTTTCAACAACGGATCTCTTGGTTCTGGCATCGATGAAGAACGCAGCGAAATGCGATAAGTAATGTGAATTGCAGAATTCA
JAAVMX010000017 9495 ----------------------------------------------------------------------------------------------------
LKHE01000582 2274 ----------------------------------------------------------------------------------------------------
ANOV01021709 1038 ----------------------------------------------------------------------------------------------------
LWBQ01000008 991939 ----------------------------------------------------------------------------------------------------
GT2 MG770309 245 -----------------------------------------------------------GA--------- C----------------------------
GT3 HM595984 175 ----------------------------------------------------------------------------------------------------
GT4 AB067744 197 ----------A---T--------T--T------------A---T-A----TA--A----------------A-------------A-----T------T-
GT5 AB067740 201 ----------A---T--------T---------------A-----A----TA--A--A---------------A---------A-A----A-------T-
GT6 EU555436 115 ----------A---T--------T---------------A-----A--------A-------T--TA----------------A-A----A-------T-
GT15 KT232017 178 --------------T--------T--T------------A---T-A----TA--A----------------A-------------A-----T------T-
GT16 KT232019 178 ----------A---T--------T---------------A-----A--------A--A---------------A---------A-A----A-------T-
GT17 KT232010 178 ----------A---T--------T---------------A-----A--------A--A---------------A---------A-A----A-------T-
 301 HsATp2/HsATp3 (RC) 400
GT1 AB067721 301 GTGAACCATCGAATCTTTGAACGCACATTGCGCCCGCCAGCACTCTGGCGGGCATGCCTGTCCGAGCGTCATCTCAACCCTCGAGCCCCCCGCCTCGCGG
JAAVMX010000017 9395 ----------------------------------------------------------------------------------------------------
LKHE01000582 2374 ----------------------------------------------------------------------------------------------------
ANOV01021709 1138 ----------------------------------------------------------------------------------------------------
LWBQ01000008 992039 ----------------------------------------------------------------------------------------------------
GT2 MG770309 146 ----------------------------------------------------------------------------------------------------
GT3 HM595984 275 ----------------------------------------------A-----------------------------------------------------
GT4 AB067744 297 --A---T-----------A----T-T-C-A-------T--------A----------------------TACCTTAACCCTTAAGCCCC-----------
GT5 AB067740 301 --A---T-----------A------T---A-------T--------A------------A---------TATCTTAACCCTCGAGCCCC-----------
GT6 EU555436 215 --A---------------A----T-T---A-------T--------A----------------------T----T--------------------TA---
GT15 KT232017 278 --A---T-----------A----T-T---A-------T--------A----------------------T----T-------------------------
GT16 KT232019 278 --A---T-----------A----T-T---A-------T--------A----------------------T-C--T------TA-----------------
GT17 KT232010 278 --A---T-----------A------T-----------T--------A------------A---------T----T-------------------------
 401 Hsprp3 (RC) 500
GT1 AB067721 401 CGGCGGGGCCCGGCCTTGGGGGTCACGGCCCCGCGCCGCCCCCTAAACGCAGTGGCGACCCCGCCGCGGCTCCCCTGCGCAGTAGCTCGCTGAG AACCT
JAAVMX010000017 9295 ---------------------------------------------------------------------------------------------- -----
LKHE01000582 2474 ---------------------------------------------------------------------------------------------- -----
ANOV01021709 1238 ---G------------------------------------------------------------------------------------------ -----
LWBQ01000008 992139 ---------------------------------------------------------------------------------------------- -----
GT2 MG770309 46 ----------------------------------------------
GT3 HM595984 375 --A----------------------------T---------------T------------------------------------------C---G-----
GT4 AB067744 397 -----------------A--------------A--------------------A-T--------------------A----A-----TA--A-- -----
GT5 AB067740 401 ---------T-------A-----T------------------T------T---A----------------------A--TG-------A-C--- -----
GT6 EU555436 315 ---------T-------A-----T------------------T----------A-------T----TA--------ATAT-------TA-C--- -----
GT15 KT232017 378 ---------T-------A-----T-------------------------T---A----------------------A--T-A------A-C--- -----
GT16 KT232019 378 -----------------A--------------A--------------------A------------------T---A----A-----TA--A-- -----
GT17 KT232010 378 ---------T-------A-----T-------------------------T---A----------------------A--T-A------A-C--- -----
 501 ITS4(RC)600
GT1 AB067721 500 CGCACCGGGAGCGCGGAGGCGGTCACGCCGTGAAACCACCACACCCTCCAGTTGACCTCGGATCAGGTAGGGATACCCGCTGAACTTAAGCATATCAATA
JAAVMX010000017 9194 ----------------------------------------------------------------------------------------------------
LKHE01000582 2573 ----------------------------------------------------------------------------------------------------
ANOV01021709 1337 ----------------------------------------------------------------------------------------------------
LWBQ01000008 992238 ---CT--C--------------------------------------------------------------------------------------------
GT2 MG770309 1
GT3 HM595984 475 --------A-------G--T----------C------------ ----------
GT4 AB067744 496 -----TA------------------------A-------T-T------TG---A---------T-----------------A------------------
GT5 AB067740 500 --------A----------------------A-----T-T--G--T-TT----A---------T-----------------A-------------T----
GT6 EU555436 414 --------A---A-------TA-A-------T-CG--T--TAG--
GT15 KT232017 477 --------A----------------------A-------T--G--T-TT----A---------T-----------------A------------------
GT16 KT232019 477 -----TA------------------------A-------T-T------T----A---C-----T-A---------------A------------------
GT17 KT232010 477 --------A----------------------A-------T--G--T-TT----A---------T-----------------A------------------
 601 686
GT1 AB067721 600 AGCGGAGGAAAAGAAACCAACAGGGATTGCCCCAGTAACGGCGAGTGAAGCGGCAACAGCTCAAATTT
JAAVMX010000017 9094 --------------------------------------------------------------------
LKHE01000582 2673 --------------------------------------------------------------------
ANOV01021709 1437 --------------------------------------------------------------------
LWBQ01000008 992338 --------------------------------------------------------------------
GT2 MG770309 1
GT3 HM595984 518
GT4 AB067744 596 ---------
GT5 AB067740 600 -----------------T--T----------TT-------------A------T--T----T------AAAATCTAGCCCCCCTACG
GT6 EU555436 467
GT15 KT232017 577 ---------
GT16 KT232019 577 ---------
GT17 KT232010 577 ---------
