# Supplementary figures and images for: Differential coexistence of multiple genotypes of Ophiocordyceps sinensis in the stromata, ascocarps and ascospores of natural Cordyceps sinensis
Source: PLoS One. 2023 Mar 9;18(3):e0270776. doi: 10.1371/journal.pone.0270776 (PMC9997936; doi:10.1371/journal.pone.0270776)

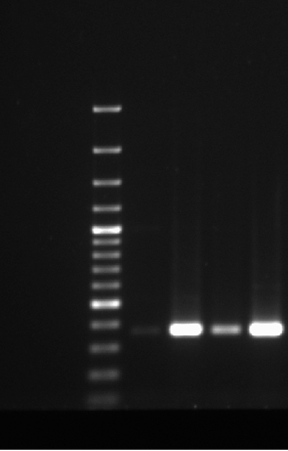

Supplement: S1 Fig — (TIF) [file pone.0270776.s002.tif]
