# Supplementary material for: Differential coexistence of multiple genotypes of Ophiocordyceps sinensis in the stromata, ascocarps and ascospores of natural Cordyceps sinensis
Source: PLoS One. 2023 Mar 9;18(3):e0270776. doi: 10.1371/journal.pone.0270776 (PMC9997936; doi:10.1371/journal.pone.0270776)
Supplement: Attachment — Submitted filename: Comments.docx [file pone.0270776.s003.docx]

*Cordyceps sinensis* is a famous-medicinal fungi, and has various function on many dieseases. This aticle reports mutiple genotype coexistence at differentially developmental stages, and has significant meaning. However, some questions need be revised.

1. Line 78-81, one sentence contains 30 references, too many.
2. Line 102-104, one sentence 33 references, too many.
3. Many sequences previously submitted in the GenBank database are wrong. Therefore, all the sequences in the last two paragraphs of results should be carefully blasted and analyzed.
4. Line 327-329, one sentence 35 references.
5. Line 332-333, too many references.
6. Discussion, the sumaries of the third part (552-553) and fourth part (568-569) aree not fully deducted on the basis of all the results.

Although this article is an meaning summary of the therapeutic values of cordycepin , i suggest the rejection of this review according to the following reasons:
