# Supplementary material for: Differential coexistence of multiple genotypes of Ophiocordyceps sinensis in the stromata, ascocarps and ascospores of natural Cordyceps sinensis
Source: PLoS One. 2023 Mar 9;18(3):e0270776. doi: 10.1371/journal.pone.0270776 (PMC9997936; doi:10.1371/journal.pone.0270776)
Supplement: Attachment — Submitted filename: 2022-9-14 Responses to the reviewers.docx [file pone.0270776.s004.docx]

**Responses to the comments from the reviewers**

**Reviewer comments:**

Reviewer #1: *Cordyceps sinensis* is a famous-medicinal fungi, and has various function on many dieseases. This aticle reports mutiple genotype coexistence at differentially developmental stages, and has significant meaning. However, some questions and the latin names need be revised.

1. Line 78-81, one sentence contains 30 references, too many.
2. Line 102-104, one sentence 33 references, too many.
3. Many sequences previously submitted in the GenBank database are wrong. Therefore, all the sequences in the last two paragraphs of results should be carefully blasted and analyzed.
4. Line 327-329, one sentence 35 references.
5. Line 332-333, too many references.
6. Discussion, the sumaries of the third part (552-553) and fourth part (568-569) aree not fully deducted on the basis of all the results.

Although this article is an meaning summary of the therapeutic values of cordycepin, i suggest the rejection of this review according to the following reasons:

Reviewer #2: In this ms, immature and mature *C. sinensis* were harvested, and mature *C. sinensis* were cultivated in laboratory to collect ascospores. Fully and semi-ejected ascospores were collected from the same sample. The molecular analyses species-/genotype-specific primers were used to analyze multiple genotypes of *Ophiocordyceps sinensis* in the stromata, ascocarps and ascospores of natural *Cordyceps sinensis*. The results indicated the metagenomic components undergo dynamic change at different development stages in a symbiotic manner, which would help to understand the development, the maturation of ascospores and artificial cultivation.

However, a number of points need to be clarified and further justified, given below,

The abstract is too simple to set out the main opinion of this paper.

Semi-ejected ascospore was considered to be one type that differ from the ejected ascospore. But be lack of the evidence in morphological and growth characteristics.

In instruction part, the author didn’t provide detailed and clear background information, and the description and the definitions are confused, e.g. the stromal fertile portion (SFP), C. sinensis, O. sinensis, natural C. sinensis, wild C. sinensis. O. sinensis fungus, the statement is wrong. Most sentences contain grammar mistakes. e.g. line 62-70, ‘ a dead Hepialidae moth larva’, should be the remains of Hepialidae moth larva, ‘intact intestine and head tissues, an intact, thick larval body cultivated insect-fungal complex’... The expressions are redundant and very difficult to read. There are many similar problems in this part.

In method part, the statement is too simple. Be deficient in descriptions of agents, materials, protocols, etc reagents, materials, protocol. And references are not fully cited.

e. g. before the extraction, how the samples were stored? How to extract?

What gene was genotype primers designed with？Whether ITS1-5.8S-ITS2 segment is suitable for all the three types primers? Why was touch-down PCR program used? ...

Line 120-121,‘ the collected samples were cultivated in our laboratory (altitude 2,200 m) in Xining City, Qinghai Province of China, with the windows left open and under conditions with sufficient watering, sunshine and ventilation.’ The culturing conditions present unclear, including light, water, ventilation, soil..., The collection method of fully/semi-ejected ascospores present unclear.

The part of result and discussion is redundant and too long, and lack a sense of reading and hierarchy. Suggested to thoroughly revise.

References

The references weren’t be completely correct and need to be formatted according to the requirement of Plos one.

Grammar mistakes,

It is very noted that this manuscript needs careful editing by expertise in English editing in pay attention to the spelling, grammar, sentence structure. Otherwise, the content can’t be clearly present. Most sentences contains grammar mistakes in this ms.

Reviewer #1 commented: “the latin names need be revised”

Reviewer #2 commented: “In instruction part, … C. sinensis, O. sinensis, natural C. sinensis, wild C. sinensis. O. sinensis fungus, the statement is wrong”

Authors’ response: Both reviewers raised concerns surrounding the taxonomy and nomenclature of natural *C. sinensis* and *O. sinensis* fungus and the Latin name usages in our manuscript. The confusion has existed since the 1840s and has extended from academy to social media and even mass markets. Unfortunately, no consensus in the Latin name(s) has been reached thus far among the taxonomists in the mycology, zoology and botany-TCM disciplines. We have attached a review paper as a reference paper to this submission pertaining to this academic confusion. We, as a group of nontaxonomy scientists, have no other choices but to temporarily use the different Latin names to differentiate between different biological identities and eagerly await a consensus from the cooperation of multidisciplinary taxonomists. The rationale for the differential usage of Latin names in our paper is listed below at the end of this section following our explanation of the Latin name confusion.

Because many relevant papers have been published in Chinese, please allow me to summarize the useful historical information herein for your reference. The review paper (attached to this submission as a reference paper) discussed this issue, which is just published in Am J Biomed Sci.

The confusion can be divided into 2 parts:

(1) **Is natural *C. sinensis* an insect-fungi complex? And whether natural *C. sinensis* = *O. sinensis* fungus?**

European scientists gave the Latin name *Cordyceps sinensis* in the 1840s specifically to the intrinsic fungus in the natural insect-fungal complex, while Prof. Edward Doubleday determined that the dead host larva belonged to Agrotis, as summarized in a systematic review by Dr. Lu D (2014). Apparently, the initial taxonomy-nomenclature studies by European scientists clearly differentiated between the natural insect-fungal complex and its intrinsic fungus. However, since then, the Latin name of the intrinsic fungus *Cordyceps sinensis* has unfortunately been used as well to refer indiscriminately to the natural insect-fungal complex.

The review paper (attached to this submission as a reference paper) includes an anatomical image of the caterpillar body of natural *C. sinensis* (Figure R1), showing that the caterpillar body of natural *C. sinensis* contains an intact larval intestine, an intact and thick larval body wall with numerous bristles, and larval head tissues, in addition to fungal mycelia. This anatomic structure ensures success in extracting larval DNA and RNA from natural *C. sinensis* and sequencing larval genes and transcripts (Zhang et al. 2014; Li et al. 2016, 2022c, 2022d).

***Figure R1. An anatomical image of the caterpillar body of natural C. sinensis.*** *“****]****” in pink indicates the thickness of the intact larval body wall. Note: this anatomic image will be published in Li et al. Molecular identification of Ophiocordyceps sinensis genotypes and the indiscriminate use of the Latin name for the multiple genotypes and the natural insect-fungi complex. Am J BioMed Sci. 2022;14(3): 115-135.*

There is no doubt that hemolymph and some of the larval soft tissues provide nutrients for the growth of infected fungi. Some mycologists often use the terms “sclerotium” and “sclerotia” to refer to the caterpillar body of natural *C. sinensis*. However, a group of TCM (traditional Chinese medicine) scientists, Ren et al. (2013), argued against the usage of the terms and suggested using the phrase “pseudosclerotic complex” to refer to the *C. sinensis* caterpillar body, based on the evidence that the caterpillar body contains not only fungal sclerotia but also an intact larval body wall and fragments of larval tissues. In addition to the intact larval body wall and fragments of larval tissues described by Ren et al. (2013), Figure R1 above shows that the *C. sinensis* caterpillar body contains an intact larval intestine, larval head tissues and numerous bristles, in addition to the massive white fungal mycelia tightly wrapped around the intact larval intestine.

Chinese mycologists Zhang et al. (2012), coauthoring and corresponding with former chairmen of the Chinese Mycological Association, Profs. Cheng-Shu Wang and Xing-Zhong Liu, agreed that the natural *C. sinensis* ≠ *O. sinensis* fungus and suggested continuously using the name *O. sinensis* for the fungus and renaming the natural insect-fungi complex using a non-Latin term: “Chinese cordyceps”. However, this renaming suggestion using the non-Latin term encountered regulatory challenges because governmental policies around the world require every natural therapeutic agent to have a unique and exclusive Latin name.

TCM scientists Ren et al. (2013) agreed that the natural *C. sinensis* ≠ *O. sinensis* fungus but disagreed with the non-Latin name usage. Instead, they suggested using the Latin name “Ophiocordyceps & Hepialidae” for natural *C. sinensis*, reflecting the anatomic structure of natural *C. sinensis* and meeting the worldwide governmental requirements. We were told in a meeting at a later time that this renaming suggestion by Ren et al. (2013) came from discussions among many TCM scientists, including senior botany-TCM taxonomists.

Apparently, the 2 groups of scientists reached a consensus on the natural *C. sinensis* ≠ *O. sinensis* fungus and expressed the necessity of renaming the natural insect-fungi complex, although they proposed different renaming suggestions. Unfortunately, they and other multidisciplinary taxonomists have not reached a consensus on an appropriate Latin name.

A senior mycologist who is a former chairman of the Chinese Mycological Association conducted a media interview using the confused Latin name. The chaotic statement spread from one newspaper to another and to the social media and mass markets, causing a negative market response with a drastic drop in the wholesale and retail prices of natural *C. sinensis* and related fermentation products. Such pure academic confusion unfortunately caused major socioeconomic turbulence affecting hundreds of thousands of innocent people in the *C. sinensis* business.

Immunologically, the anatomic structure shown in Figure R1 reflects the host’s immune defense during fungal infection. Lei and Shui (2015), Meng et al. (2015), and Wang et al. (2016, 2018) conducted mycology-ecology, metagenome, and metatranscriptome studies and reported host innate immunity and acquired immunological responses during fungal infection in ghost moth larvae. These findings and the anatomic structure shown in Figure R1 indicate that the caterpillar body of natural *C. sinensis* is completely different from the culture medium that passively provides nutrients for *in vitro* fungal growth in solid and liquid cultures and fermentations (Li et al. 2022c, 2022d).

In addition, numerous multidisciplinary studies demonstrated distinct profiles between the stroma (fungi) and caterpillar body (insect-fungi complex) of natural *C. sinensis*, including mycobiotal/microbiotal profiles, metagenomic DNA pools, RAPD random molecular marker polymorphisms, metatranscriptomic cDNA pools, metaproteomic expression polymorphisms, HPLC fingerprints of chemical constituents, distinct pharmacological functions and even opposite therapeutic effects of the 2 compartments of natural *C. sinensis* (Liu et al. 2006; Zhu et al. 2007, 2010, 2017; Xiao et al. 2009; Feng et al. 2010; Liang et al. 2010; Zhang et al. 2010, 2020; Yao et al. 2011, 2014; Dong et al. 2014; Xia et al. 2015; Li et al. 2016, 2022; Shao et al. 2019).

All of these findings prove that natural *C. sinensis* ≠ *O. sinensis* fungus and that natural *C. sinensis* and *O. sinensis* fungi represent distinct biological identities, although they are somewhat related in mycology, therapeutics and some other scientific aspects. Therefore, they should be treated scientifically discriminately and given unique and exclusive Latin names to satisfy governmental regulation requirements worldwide with respect to every natural therapeutic agent having a unique and exclusive Latin name.

(2) **Does the Latin name *O. sinensis* refer to a single fungus or a group of fungi?**

Chinese mycologists and molecular mycologists published several mycobiota and microbiota studies (Zhang et al. 2010a, 2010b; Xia et al. 2015, 2016) and reported the detection of >90 fungal species spanning >37 genera in natural *C. sinensis*. However, it was interesting that none of the 17 *O. sinensis* genotypes were detected in these studies. Metagenomic and metatranscriptomic studies confirmed the coexistence of multiple fungi in natural and cultivated *C. sinensis* (Xiang et al. 2014; Xia et al. 2017; Zhong et al. 2018; Li et al. 2019; Zhao et al. 2020). Because of the coexistence of multiple fungal species, natural *C. sinensis* should be called an insect-fungi complex or an insect-fungal complex but should not be called an insect-fungus complex. However, the correct anamorph of *O. sinensis* has not been determined because none of the intrinsic fungi meet all 4 criteria of Koch’s postulates. Thus, no definite answer can be offered to the question “Does the Latin name *O. sinensis* refer to a single fungus or a group of fungi?” Many mycologists hypothesize that *Hirsutella sinensis* is likely the correct anamorph of *O. sinensis*, while many others consider *H. sinensis* likely to be one of the anamorphs of *O. sinensis* (more discussions below). Because many papers were published in Chinese, please allow me to summarize the useful information herein for your reference.

Kinjo & Zang (2001) reported 3 phylogenetic clades of *O. sinensis* sequences that were detected from natural *C. sinensis* specimens collected from different production areas.

Stensrud et al. (2007) conducted a Bayesian phylogenetic analysis on 71 ITS sequences of *O. sinensis* available in the GenBank database at that time and reported the phylogenetic differences of GC-biased Group-A (Genotype #1) and AT-biased Groups B‒C (Genotypes #4‒5) of *O. sinensis*. They stated “Judged from the ITS phylogeny presented here, the A, B and C groups share a common ancestor … The three evolutionary lineages (A–C) of *C. sinensis* may turn out to have different medicinal effects (*i.e.,* secondary metabolites) … Groups B and C … showed highly accelerated evolution of the 5.8S gene compared with group A … Such a large sequence variation of the 5.8S nrDNA far exceeds what is normally observed in fungi (and other organisms), even at higher taxonomic levels (genera and family).” This is the first paper indicating that *O. sinensis* refers to 3 phylogenetic groups of fungi, which are taxonomically distinct “at higher taxonomic levels (genera and family)” through “highly accelerated evolution” from “a common ancestor”.

Xiao et al. (2009) used genotype-specific primers and nested PCR techniques and reported inconsistent cooccurrence of the 3 groups of *O. sinensis* genotypes in natural *C. sinensis*. They thus concluded that the mutant sequences of *O. sinensis* likely belonged to different fungi.

Wei et al. (2016) (corresponding with the honorary chairman of the Chinese Mycological Association and a senior member of the Chinese Academy of Sciences) reported the detection of a sole teleomorphic GC-biased Genotype #1 (Group A) (green arrow in Figure R2; a screen capture of Fig 6 of Wei et al. 2016, with addition of side notes) in natural *C. sinensis* and a sole teleomorphic AT-biased Genotype #4 (Group B) (purple arrows in Figure R2) in cultivated *C. sinensis*, indicating the existence of at least 2 teleomorphs of *O. sinensis*, which are taxonomically distinct “at higher taxonomic levels (genera and family)” (Stensrud et al. 2007).

### ***Figure R2. Screen capture of Fig 6 (a Maximum Likelihood phylogenetic tree that was conferred using software Mega5) from Wei et al. 2016 with addition of side notes.*** *The* ***purple arrows*** *and a* ***green arrow*** *indicate the teleomorphic O. sinensis of cultivated and natural C. sinensis, respectively.* *The sequences of teleomorphic O. sinensis in Fruiting body 2 and Mycelia 3 of cultivated C. sinensis were clustered to the AT-biased Genotype #4 clade (side-noted in* ***red****). The sequences of teleomorphic O. sinensis in natural C. sinensis G3 and the 3 anamorphic H. sinensis strains (H01‐20140924‐03, 130508‐KD‐2B, and 20110514) that were used as inoculants in this artificial cultivation project were clustered to the GC-biased Genotype #1 clade (side-noted in* ***blue****).*

Wei et al. (2016) also showed that AT-biased Genotype #4 of *O. sinensis* is culturable, as evidenced by sample “Mycelia 3” (one of the purple arrows in Figure R2) obtained from cultures of the caterpillar body of cultivated *C. sinensis*. Similarly, Mao et al. (2013) also cultured the tissues of natural *C. sinensis* specimens collected from geographically distant production areas and obtained sequences of AT-biased Genotype 4 or 5 but not GC-biased Genotype #1. However, none of the studies obtained pure cultures of AT-biased *O. sinensis* genotypic fungi with *H. sinensis*-like morphology for multigene analysis and whole genome sequencing.

Wei et al. (2016) also demonstrated a species contradiction between the 3 inoculants (anamorphic GC-biased Genotype #1 *H. sinensis* Strains H01‐20140924‐03, 130508‐KD‐2B, and 20110514) that were reportedly used in the artificial inoculation project and the teleomorphic fungus of AT-biased Genotype #4 of *O. sinensis* in the final cultivated *C. sinensis* product (Figure R2). According to the fourth criterion of Koch’s postulates, the species contradiction invalidated the sole anamorph hypothesis for Genotype #1 *H. sinensis*, which was postulated 10 years ago by the same group of key authors (Wei et al. 2006).

Wei et al. (2006) (corresponding with the honorary chairman of the Chinese Mycological Association and a senior member of the Chinese Academy of Sciences) proposed the sole anamorph hypothesis for *H. sinensis* and concluded that *Hirsutella hepiali*, *Cephalosporium dongchongxiacaonis*, and *Synnematium sinense* are synonyms of *H. sinensis*. Actually, the authors only studied *S. sinense* strains S1, S2, S3, and S4, *H. sinensis* strain G2, and *C. sinensis* strain G3. The synonym conclusion is debatable with respect to whether the sequences under the 4 names truly belong to the same fungus for the following 2 reasons.

(i) The synonym conclusion was solely based on the previous identification of *H. sinensis*-like morphologic and growth characteristics of *C. dongchongxiacaonis*, *H. hepiali*, *H. sinensis*, and *S. sinense* without evidence from molecular genotyping, multigene analysis or genome sequencing. However, studies reported that mutant genotypes of *O. sinensis* have the same *H. sinensis*-like morphologic and growth characteristics but are different taxonomically “at higher taxonomic levels (genera and family)” (Stensrud et al. 2007; Xiao et al. 2009; Mao et al. 2013; Wei et al. 2016).
(ii) Wei et al. (2006) designed random amplified polymorphic DNA (RAPD) experiments with biased selection of only 5 random primers, and a density-unweighted UPGMA algorithm was inappropriately selected for the data analysis (Ni et al. 2014; Yao et al. 2014; Li et al. 2016, 2022; Zhu & Li 2017). Wei et al. (2006) analyzed the ITS sequences of the strains of *S. sinense*, *H. sinensis* and *C. sinensis* and published a phylogenetic tree that was inferred using the neighbor-joining phylogenetic algorithm.

Apparently, the computational biological methods used by Wei et al. (2006) were inappropriate; consequently, the results were debatable. UPGMA and neighbor-joining algorithms are not the most reliable means for phylogenetic construction and are almost never acceptable in top-tier journals. Ni et al. (2014) and Yao et al. (2014) reported sufficient data with using 20 random primers and proved that Wei et al. (2006) biasedly selected only 5 random primers, leading to the published results questionable. Ni et al. (2014) and Yao et al. (2014) also proved that the density-unweighted UPGMA algorithm used by Wei et al. (2006) was inappropriate in inferring a phylogenetic tree due to missing significant molecular information buried in RAPD DNA bands. This algorithm does not usually produce a topology that fulfils the minimum evolution or least-squares criterion. The neighbor-joining algorithm is a computational shortcut to quickly infer a phylogenetic tree based on genetic distance and is most vulnerable to missing data. Computational biology scientists recommend using a Bayesian algorithm. Thus, Wei et al. (2006) actually left a serious question of whether the fungal sequences under the 4 names truly belong to the same *H. sinensis* fungus. However, other scientists are unable to repeat the experiments or sequence analysis because of unavailability of the original fungal strains under these names and because Wei et al. (2006) did not publish the ITS sequences of these fungal strains in their paper or upload the sequences to GenBank.

Engh (1999) (a graduate student at the University of Oslo) reported the molecular identification of the *Cordyceps-Tolypocladium* complex, the sequences of which were uploaded to GenBank by Stensrud et al. (2005). The “*Cordyceps*” sequence discovered by Engh (1999) is *C. sinensis* AJ786590, and the “*Tolypocladium*” sequence is *T. inflatum* AJ786599. Stensrud et al. (2007) further analyzed the sequences and reported that AJ786590 belonged to AT-biased Group B (Genotype #4). Barseghyan et al. (2011) (coauthoring with Prof. Solomon P. Wasser) conducted macro- and micromycology experiments and concluded that *H. sinensis* and *Tolypocladium sinensis* were the anamorphs of *O. sinensis*, proposing a dual anamorph hypothesis without molecular genotyping of the *C. sinensis* fungus.

Zhu et al. (2012) summarized the literature findings and put together “at least 6 mutant genotypes of *O. sinensis*”, which were discovered in natural *C. sinensis*, including GC-biased Genotypes #1‒3 and AT-biased Genotypes #4‒6 of *O. sinensis*. These 6 genotypes have been confirmed as correct *O. sinensis* sequences by Stensrud et al. (2007) and Zhang et al. (2009, 2013). Genotypes #2‒6 contain multiple scattered transition point mutations (Figure R3).

Chinese mycologists Zhang et al. (2013) (“On the reliability of DNA sequences of *Ophiocordyceps sinensis* in public databases. ***J Ind Microbiol Biotechnil***. 2013;40: 365−378”; corresponding with a former chairman of the Chinese Mycological Association) analyzed 397 ITS sequences using the minimum evolutionary (ME) algorithm. The topology of the ME phylogenetic tree is consistent with those of the Bayesian phylogenetic trees reported by Stensrud et al. (2007) and by us (*cf*. Fig 7 of our manuscript PONE-D-22-17339). These 2 studies confirmed that the transition mutants in Groups A‒C (*i.e.*, Genotypes #1 and #4‒5) were correct sequences of *O. sinensis*, while other sequences in Groups D‒E (Stensrud et al. 2007) and Groups D‒G (Zhang et al. 2013) belonged to other fungi. However, Zhang et al. (2013) did not discuss the ITS sequence HM595984 (Genotype #3) that was discovered by their own group (Zhang et al. 2009) from 2 natural *C. sinensis* specimens collected from Nyingchi of Tibet. Zhang et al. (2009) believed that GC-biased Genotype #3 represented an ancestor strain of *H. sinensis* and that Nyingchi of Tibet is the center of the evolutionary origin of the *O. sinensis* genus under the geography of *O. sinensis* evolution.

**Figure R3. ITS sequence alignment of the transition mutant genotypes of O. sinensis** (adapted from Li et al. 2022 14(3): (in press)). The sequences contain complete or partial segments of ITS1-5.8S-ITS2 rDNA and partial segments of the 18S and 28S genes. “GT” stands for the O. sinensis genotype. AB067721, MG770309, and HM595984 represent GC-biased Genotypes #1‒3 of O. sinensis, respectively. AB067744, AB067740, EU555436, KT232017, KT232019, and KT232010 represent AT-biased Genotypes #4‒6 and #15‒17 of O. sinensis, respectively. ANOV01021709, JAAVMX010000017, LKHE01000582, LWBQ01000008, and NGJJ01000799 are the ITS1-5.8S-ITS2 rDNA segments of the genome assemblies of Genotype #1 H. sinensis strains Co18, IOZ07, 1229, ZJB12195, and CC1406-203, respectively. Hyphens indicate identical bases, and spaces denote unmatched sequence gaps.

**Figure R4. ITS sequence alignment of the 5 groups of O. sinensis transversion mutants and 2 insertion/deletion mutants of O. sinensis compared with AB067721** (adapted from Li et al. 2016b). AB067721 represents Genotype #1, the GC-biased O. sinensis, as the reference sequence. AJ488254 (RC, reverse complement), GU246286, GU246288, GU246287, and JQ695935 represent Genotypes #7, #8, #9, #10, and #11, the O. sinensis transversion mutants. GU246296 and GU246280 represent Genotype #12, the insertion/deletion mutants of O. sinensis. The residues in red were annotated in GenBank as 18S (5’ end) or 28S (3’ end) rRNA; the residues in green were annotated as ITS1; the residues in pink were annotated as 5.8S rRNA; and the residues in blue were annotated as ITS2. Hyphens indicate identical bases, and spaces denote unmatched sequence gaps.

Li et al. (2016) checked the phylogenetic results reported by Zhang et al. (2013) and found that Group A of *O. sinensis* contained several variable ITS sequences harboring multiple scattered transversion and/or insertion/deletion alleles, which were distinct from Genotype #1 sequences in Group A and should be treated differently. Li et al. (2016) then reported a total of 12 genotypes of *O. sinensis*. In addition to the 6 transition point mutant genotypes shown in Figure R3, the 6 additional GC-biased genotypes include Genotypes #7‒11 with multiple scattered transversion point mutations and Genotype #12 with multiple scattered insertion/deletion point mutations (Figure R4; Table R1). These additional GC-biased genotypes were analyzed as correct sequences of *O. sinensis* in Group A by Zhang et al. (2013).

### Table R1 Segmented sequence similarities of the ITS1, 5.8S and ITS2 sequences of the GC-biased AB067721 (Genotype #1) and Genotypes #7‒12 of *O. sinensis*.

|  | Genotype | AT content (%) of total | Percent similarity *vs.* Genotype #1 AB067721 | | | |
| --- | --- | --- | --- | --- | --- | --- |
|  |  |  | ITS1 | 5.8S | ITS2 | ITS1-5.8S-ITS2 |
| AJ488254 | #7 | 34.7% | 93.2% | 98.7% | 89.4% | 93.9% |
| GU246286 | #8 | 40.5% | 86.2% | 94.8% | 87.9% | 89.6% |
| GU246288 | #9 | 35.6% | 96.3% | 98.7% | 91.5% | 95.3% |
| GU246287 | #10 | 40.2% | 86.2% | 92.9% | 72.4% | 83.2% |
| JQ695935 | #11 | 43.7% | 94.3% | 100% | 55.1% | 81.6% |
| GU246296 | #12 | 36.3% | 99.4% | 99.4% | 87.0% | 94.9% |

*Note: [Modified from Table 2 of Li et al. (2016)].*

As reported herein in our manuscript (PONE-D-22-17339), we discovered additional GC-biased Genotypes #13‒14 (Figure R4) in the semi- and fully ejected ascospores of natural *C. sinensis* and AT-biased Genotypes #15‒17 with multiple scattered transition point mutations (Figure R3). Thus, there are a total of 17 *O. sinensis* genotypes that have been discovered thus far, among which only Genotype #1 *H. sinensis* has been taxonomically identified, and its genome has been sequenced. No publication thus far reports taxonomic identifications of pure cultures for Genotypes #2‒17 of *O. sinensis*.

There are several special issues relating to Latin name(s) usage:

(A)	The renaming study: Sung et al. (2007) renamed *Cordyceps sinensis* to *Ophiocordyceps sinensis* using the *Hirsutella sinensis* Strain EFCC 7287 (GC-biased Genotype #1) as the nomenclature study reference for multigene analysis without expanding their nomenclature project to the remaining 16 genotypes (Zhu et al. 2018). The phylogenetic relationship of GC- and AT-biased mutant sequences was recognized scientifically almost at the same time (Stensrud et al. 2007), although earlier discovery of the variable sequences was reported by Engh (1999), Kinjo and Zang (2001), and Stensrud et al. (2005). There have been no reports on the purification of Genotypes #2‒17 for multigene analysis or whole genome sequencing.

Thus, because the nomenclature standard was Genotype #1 *H. sinensis* for renaming *C. sinensis* to *O. sinensis* (Sung et al. 2007), this renaming should not affect the name “natural *C. sinensis*”, which has been indiscriminately used for the insect-fungi complex for 170^+^ years.

(B)	The “ITS pseudogene” hypothesis: Li et al. (2013) opposed the notion that multiple *O. sinensis* genotypes represent independent fungi (Stensrud et al. 2007; Xiao et al. 2009). Li et al. (2013) reported coculture of GC-biased Genotype #1 and AT-biased Genotype #5 in 8 of 15 clones (Group 1) after 25-day liquid incubation of mono-ascospores of natural *C. sinensis*, but only Genotype #1 in the remaining 7 clones (Group 2) and no pure culture of Genotype #5. They hypothesized with overinterpretation that the sequences of ALL AT-biased genotypes were the “ITS pseudogene” components of “a single genome” of GC-biased Genotype #1, although they did not detect sequences of AT-biased Genotypes #4, #6, and #15‒17 in the ascosporic cultures.

This “ITS pseudogene … in a single genome” hypothesis failed to explain the findings from their own study: distinct *O. sinensis* genomes of 8 heterogeneous clones (Group 1) containing the so-called “ITS pseudogene” and 7 homogenous clones (Group 2) containing no “ITS pseudogene”. Therefore, the 2 populations of “pure” cultures from mono-ascospores suggest genetic heterogeneity of Genotype #1 *H. sinensis* harboring distinct genomes. Strain 1229 (one of the homogenous Group 2 strains) has been used for genome sequencing, obtaining the genome assembly LKHE00000000 (Li et al. 2016). However, there has been no report of the genome sequences of Group 1 strains 10 years after the initial discovery, although genome sequencing of the heterogeneous Group 1 strains would provide critical evidence to support the hypothesis of “ITS pseudogene … in a single genome”.

The key authors of (Li et al. 2013) and other coauthors published another paper (Li et al. 2020b) and further hypothesized that all AT-biased genotypes are “ITS pseudogene” components that were induced through RIP (repeat-induced point mutations) mutagenesis within “a single genome” after a new GC-biased “ITS haplotype was generated”.

Zhu et al. (2019) and Li et al. (2020a; especially its Appendix A) discussed in detail the “ITS pseudogene” hypothesis by carefully analyzing the study design and results reported by Li et al. (2013). They found that Li et al. (2013) provided incomplete and controversial evidence backing up the “ITS pseudogene” hypothesis. Importantly, the sequences of AT-biased genotypes are absent in the genome assemblies ANOV00000000, [JAAVMX000000000](https://www.ncbi.nlm.nih.gov/nucleotide/JAAVMX010000002.1?report=genbank&log$=nuclalign&blast_rank=6&RID=D8E2V31A01R), LKHE00000000, LWBQ00000000, and NGJJ00000000 of GC-biased Genotype #1 *H. sinensis* Strains Co18, IOZ07, 1229, ZJB12195, and CC1406-203, respectively (Hu et al. 2013; Li et al. 2016; Jin et al. 2020; Liu et al. 2020; Shu et al. 2020). Apparently, genome sequence analysis has proven the genomic independence of the *O. sinensis* genotypes, which is not supportive of the notion that AT-biased *O. sinensis* genotypes were induced through RIP mutagenesis within “a single genome” after a new GC-biased “ITS haplotype was generated” (Li et al. 2013, 2019, 2020b).

Further genome analysis identified multiple repetitive ITS copies in the genome assemblies [JAAVMX000000000](https://www.ncbi.nlm.nih.gov/nucleotide/JAAVMX010000002.1?report=genbank&log$=nuclalign&blast_rank=6&RID=D8E2V31A01R) and NGJJ00000000 of Genotype #1 *H. sinensis* Strains IOZ07 and CC1406-203, respectively (Liu et al. 2020; Shu et al. 2020). (Note: the genome assemblies ANOV00000000, LKHE00000000 and LWBQ00000000 of *H. sinensis* Strains Co18, 1229 and ZJB12195, respectively, contain only one copy of ITS sequences, indicating that other repetitive ITS copies might have been discarded during assembly of genome shotgun sequences.) The multiple repetitive ITS copies in [JAAVMX000000000](https://www.ncbi.nlm.nih.gov/nucleotide/JAAVMX010000002.1?report=genbank&log$=nuclalign&blast_rank=6&RID=D8E2V31A01R) and NGJJ00000000 contain multiple scattered insertion/deletion and transversion point mutations, which theoretically might not be induced through RIP mutagenesis that causes cytosine-to-thymine (C-to-T) and guanine-to-adenine (G-to-A) transition mutations. Perhaps the transition mutant AT-biased genotypes were indeed induced through RIP mutagenesis (or through other mutagenic mechanisms) from a common evolutionary ancestor in the long history of evolution (Stensrud et al. 2007) and became independent evolutionary offspring *O. sinensis* fungi. There has been no evidence to prove that the AT-biased genotypes with multiple scattered transition point mutations shown in Figure R3 emerged after a new GC-biased “ITS haplotype was generated” and cooccurred “in a single genome” of Genotype #1 of *O. sinensis*, as misjudged by Li et al. (2020b).

Many other studies have demonstrated the differential cooccurrence of multiple genotypes in different combinations in different compartments of natural *C. sinensis* (Xiao et al. 2009; Zhu et al. 2010, 2012; Gao et al. 2011; Yao et al. 2011; Li et al. 2016b, 2022). The abundance of naturally co-occurring, multiple GC- and AT-biased genotypes underwent dynamic alterations in an asynchronous and disproportional manner during the maturation of natural *C. sinensis* (Zhu et al. 2010, 2012, 2019; Gao et al. 2011, 2012; Yao et al. 2011; Li et al. 2016b, 2020a, 2022). These findings further confirm the genomic independence of multiple *O. sinensis* genotypes, the sequences of which belong to independent *O. sinensis* fungi as the result of a long history of evolution (Stensrud et al. 2007; Xiao et al. 2009; Zhu and Wu 2015; Zhu and Li 2017; Li et al. 2022) but are not supportive of the “ITS pseudogene … in a single genome” hypothesis.

(C)	Implementation of IMA’s nomenclature rule: based on the sole anamorph hypothesis for *H. sinensis* proposed by Wei et al. (2006), mycologists Zhang et al. (2013) suggested implementing the “One Fungus=One Name” nomenclature rule in *O. sinensis* research. Some papers followed this suggestion. However, this implementation suggestion ignored the fact that the GC-biased Genotype #1 *H. sinensis* Strain EFCC 7287 was used as the renaming reference by Sung et al. (2007) without expanding the nomenclature project to Genotypes #2‒17. The taxonomic positions of Genotypes #2‒17 have not been determined. Based on findings of the multiple genomically independent *O. sinensis* genotypes, it is thus questionable whether it is appropriate to implement the nomenclature rule in *O. sinensis* research because the prerequisite of the rule states that it must be “One Fungus” before implementation. Apparently, ensuring that *O. sinensis* is “One Fungus” has not succeeded; instead, the multiple genotypes are genomically independent and likely belong to independent *O. sinensis* fungi (Stensrud et al. 2007; Xiao et al. 2009).

Let us use real cases to explain the confusion. Prof. Zheng Yu-Guo and his team published 2 important papers entitled [79] “Transcriptome sequencing and analysis of the entomopathogenic fungus *Hirsutella sinensis* isolated from *Ophiocordyceps sinensis*” in BMC Genomics in 2015, and [54] “Genome sequencing and analysis of the entomopathogenic fungus *Hirsutella sinensis* isolated from *Ophiocordyceps sinensis*” in AMB Express in 2020. Replacing the anamorphic name *Hirsutella sinensis* with the teleomorphic name *Ophiocordyceps sinensis* when following the suggestion of (Zhang et al. 2013) and implementing the nomenclature rule “One Fungus=One Name” would unfortunately produce the illogical “*Ophiocordyceps sinensis* isolated from *Ophiocordyceps sinensis*” in the above paper titles. Would scientists and readers of scientific journals accept such titles of scientific papers? Additionally, it is not scientifically legitimate to state that the fungus “*Hirsutella sinensis*” was isolated from fungus “*Ophiocordyceps sinensis*”, when Sung et al. (2007) used *H. sinensis* strain EFCC 7287 as the nomenclature reference and renamed *Cordyceps sinensis* to *Ophiocordyceps sinensis*. Obviously, a different level of confusion regarding Latin name usage is present in the titles indicating that *Ophiocordyceps sinensis* fungus = natural *C. sinensis* insect-fungi complex.

By aggregating the above analysis, the answer to the second question is that the Latin name *O. sinensis* most likely refers to a group of *O. sinensis* fungi that harbor *H. sinensis*-like morphological and growth characteristics. Thus, it is not appropriate, at least at this time, to implement the IMA’s nomenclature rule of “One Fungus=One Name” in *O. sinensis* studies before the taxonomic positions of Genotypes #2‒17 of *O. sinensis* have been determined (Zhu et al. 2018).

Both reviewers raised the academic controversy regarding the Latin name confusion relating to natural *C. sinensis* and *O. sinensis* fungi. The controversy is quite complicated to us as a group of nontaxonomy scientists, while each side of taxonomists in mycology, zoology, and botany-TCM has stood strongly on their own beliefs, and no consensus has been reached thus far. Apparently, the reviewers of our manuscript also hold a strong belief. Unfortunately, we are not among the taxonomists and are not capable of making a judgment call to the confusion.

In this confusing situation with many academic uncertainties, we made the following temporary decisions regarding Latin name usage in our manuscript:

(i). Following the suggestion raised by mycologists Zhang et al. (2012, corresponding with the senior member and former chairman of the Chinese Mycological Association), ***O. sinensis* is used to refer to the intrinsic fungus/fungi** in the natural insect-fungi complex;
(ii). For the Latin name for **natural and cultivated insect-fungi complexes**, Chinese mycologists and TCM scientists proposed different suggestions, each of which poses its own shortcomings (*cf*. the attached review paper). It is not easy for us to make a choice regarding using either “the non-Latin term Chinese cordyceps” or “Ophiocordyceps & Hepialidae”.

Fortunately, after consulting with many taxonomists in mycology and botany-TCM, Profs. Yong-Jie Zhang and Xing-Zhong Liu (the senior member and former chairman of the Chinese Mycological Association) suggested in a scientific meeting the use of the term “natural *C. sinensis*” for natural insect-fungi complexes. We then follow this suggestion and continue customarily using the old name “natural *C. sinensis*”, which has been used for 170^+^ years;

(iii). Retention of the use of the old **anamorphic name *H. sinensis*** for **Genotype #1** of *O. sinensis* to distinguish it from the rest of the *O. sinensis* genotypes for 3 reasons:

(a) The multiple genotypes of *O. sinensis* showed genomic independence and thus most likely belong to independent fungi (Stensrud et al. 2007; Xiao et al. 2009);

(b) Wei et al. (2016, corresponding with the honorary chairman of the Chinese Mycological Association and a senior member of the Chinese Academy of Sciences) reported at least 2 teleomorphs of *O. sinensis* (*cf*. Figure R2); and

(c) Implementing IMA’s nomenclature rule “One Fungus=One Name” in *O. sinensis* studies is questionable before taxonomically determining that the name *O. sinensis* truly refers to a single fungus “One Fungus”;

(iv). No Latin names have been specifically given to **Genotypes #2‒17** that show genomic independence and using *O. sinensis* for them is the only option.

Again, as we stated in our manuscript, the Latin name usage in our manuscript is temporary and hopefully will be replaced by unique and exclusive Latin names. We are not taxonomists and are patiently awaiting a consensus from a cooperative study by multidisciplinary taxonomists. We hope that the academic editor and reviewers will consider the current situation of academic confusion and the difficulty that all of us are facing.

Reviewer #1 commented: “Line 78-81, one sentence contains 30 references, too many. Line 102-104, one sentence 33 references, too many. Line 327-329, one sentence 35 references. Line 332-333, too many references.”

Authors’ response: Following the reviewer’s comments, we deleted many references in the following sentences.

“Line 78-81” Now Lines 94‒98: deleted 9 references.

“Line 102-104” Now Lines 117‒121: deleted 7 references.

“Line 327-329”: The whole paragraph has been deleted.

“Line 332-333” Now Lines 480‒481: deleted 13 references.

Reviewer #1 commented: “Many sequences previously submitted in the GenBank database are wrong. Therefore, all the sequences in the last two paragraphs of results should be carefully blasted and analyzed.”

Authors’ response: Yes, the Reviewer is correct. The GenBank database contains at least 668 ITS sequences under the name of *C. sinensis* or *O. sinensis* and under GenBank taxid: 72228. The sequences have been thoroughly analyzed by molecular mycologists and by us. One of the key references is as follows:

Stensrud Ø, Schumacher T, Shalchian-Tabrizi K, Svegardenib IB, Kauserud H. Accelerated nrDNA evolution and profound AT bias in the medicinal fungus *Cordyceps sinensis*. ***Mycol. Res***. 2007;111: 409–415.

Stensrud et al. (2007) conducted Bayesian phylogenetic analysis on 71 ITS sequences and concluded that “Judged from the ITS phylogeny presented here, the A, B and C groups share a common ancestor …The three evolutionary lineages (A–C) of *C. sinensis* may turn out to have different medicinal effects (*i.e.,* secondary metabolites) … Groups B and C … showed highly accelerated evolution of the 5.8S gene compared with group A … Such a large sequence variation of the 5.8S nrDNA far exceeds what is normally observed in fungi (and other organisms), even at higher taxonomic levels (genera and family).” Meanwhile, they concluded that sequences in Groups D and E belong to other fungi.

Another key phylogenetic study is (Zhang et al. 2013; corresponding with the senior member and former chairman of the Chinese Mycological Association), which analyzed 397 ITS sequences available in GenBank using the minimum evolutionary algorithm. The topology of the ME phylogenetic tree is consistent with that of the Bayesian phylogenetic tree (Stensrud et al. 2007). These 2 phylogenetic studies confirmed that the transition mutation sequences in Groups A‒C (*i.e.*, Genotypes #1 and #4‒5) were correct sequences of *O. sinensis*, while other sequences in Groups D‒E (Stensrud et al. 2007) and Groups D‒G (Zhang et al. 2013) belonged to other fungi. However, Zhang et al. (2013) did not discuss the ITS sequence HM595984 (Genotype #3; more discussion below), which was discovered by the same group (Zhang et al. 2009).

In our manuscript, we excluded those sequences in Groups D−G, except for several sequences for the AB067719-type Group-E fungus, which is one of the parental fungi of the offspring Genotypes 13−14 of *O. sinensis*. Fig 7 of our manuscript shows a Bayesian phylogenetic tree for 17 genotypes of *O. sinensis*, in which a few sequences of the AB067719-type Group-E fungus were included and used as an “outgroup control”. Both Stensrud et al. (2007) and Zhang et al. (2013) clustered the AB067719-type sequences in Group E, belonging to different fungi other than *O. sinensis*, although these sequences were incorrectly annotated as “*C. sinensis*” under GenBank taxid: 72228.

We certainly agree with the judgment made by Stensrud et al. (2007) and Zhang et al. (2013) regarding the Group E fungus. The AB067719-type sequences were included in the Bayesian analysis as the outgroup control because the fungus genetically relates to Genotypes #13‒14 of *O. sinensis*, as reported in our manuscript (*cf*. Table 3 and Fig 6). Other than the outgroup control AB067719-type sequences, all other sequences in Fig 7 belong to the 17 genotypes of *O. sinensis* (*cf*. Figures R3 and R4 above), regardless of whether they are intraspecific variants within a single genus (Kinjo & Zang 2001) or interspecific variations “at higher taxonomic levels (genera and family)”, as stated by Stensrud et al. (2007).

Reviewer #1 commented: “Discussion, the sumaries of the third part (552-553) and fourth part (568-569) aree not fully deducted on the basis of all the results.”

Authors’ response:

(1) “Discussion, … third part (552-553)”: **Genotypes #4 and #15 of AT-biased Cluster-B play special roles in the early development of stroma and SFP (with ascocarps)**. This is now in Line 707.

This section discusses the information of differential occurrence of genotypes within AT-biased Cluster-B as shown in Table 2 (Lines 388‒390) and Fig 7 (Lines 448‒456) of our paper. The differential occurrence of the *O. sinensis* genotypes may be related to the maturation stages of the study materials and to different compartments of the samples tested (*cf*. Table 2), demonstrating the genomic independence of various genotypes.

We noticed that some published papers reported the detection of only Genotype #4 or Genotype #5 of *O. sinensis* in natural and cultivated *C. sinensis* without the cooccurrence of Genotype #1 or other genotypes (Engh 1999; Kinjo and Zang 2001; Mao et al. 2013; Wei et al. 2016). Our results and the discussion in this section remind scientists to pay close attention to the maturation stage of the study samples, whether other *O. sinensis* genotypes may cooccur in lower abundance, whether different compartments of natural *C. sinensis* should be examined additionally, and whether there is a need to combine other molecular techniques.

We have changed the title of this section to **“Differential occurrence of Genotypes #4 and #15 of AT-biased Cluster-B”** and slightly modified the 2^nd^ paragraph by adding “(*cf.* Fig 7)”.

(2) “Discussion … fourth part (568-569)”: **Fungal factors involved in the control of the development, maturation and ejection of *C. sinensis* ascospores**. This is now in Lines 723‒724.

This section summarizes the information shown in Tables 2‒3 and Figures 5‒6 of our manuscript, including the occurrence of GC- and AT-biased genotypes of *O. sinensis*, *S. hepiali*, and the AB067719-type fungus in the ascospores of natural *C. sinensis*. This section focuses on the fungal factors detected in the *C. sinensis* ascospores and discusses the possible functions of the fungal factors symbiotically controlling the development, maturation and ejection of *C. sinensis* ascospores.

We deleted a paragraph in Lines 570‒574 and several sentences in Lines 596‒601 in the original manuscript submitted last time. This paragraph and the sentences discussed the results presented in the companion paper [69] “Li Y-L, Li X-Z, Yao Y-S, Wu Z-M, Lou Z-Q, Xie W-D, et al. Altered GC- and AT-biased genotypes of *Ophiocordyceps sinensis* in the stromal fertile portions and ascospores of natural *Cordyceps sinensis*”. We will leave the discussions pertaining to these results to the companion paper.

Reviewer #1 commented: “Although this article is an meaning summary of the therapeutic values of cordycepin, i suggest the rejection of this review according to the following reasons:”

Authors’ response: We do not quite understand the comments “this article is an meaning summary of the therapeutic values of cordycepin” and “this review”.

First, our paper is not a review paper but a research paper that reports many new findings in morphology and molecular mycology. Second, our paper does not directly pertain to “cordycepin”. Cordycepin has been detected in other *Cordyceps* species, such as *C. militaris*, but not in the fungus *O. sinensis*, which has been reported by many studies in the past. Please see the following paper corresponding with Prof. Cheng-Shu Wang, the senior member and former chairman of the Chinese Mycological Association.

Xia Y-L, Luo F-F, Shang Y-F, Chen P-L, Lu Y-Z, Wang C-S. Fungal Cordycepin Biosynthesis Is Coupled with the Production of the Safeguard Molecule Pentostatin. ***Cell Chem Biol***. 2017;24: 1479–1489. [http://dx.doi.org/10.1016/j.chembiol.2017.09.00](%20http://dx.doi.org/10.1016/j.chembiol.2017.09.00)1

However, other naturally cooccurring fungi in natural *C. sinensis* may produce cordycepin, such as *Samsoniella hepiali* (synonym *Paecilomyces hepiali*) (cf. Table 2 and Fig 5 of our manuscript). Please see the following transcriptome study paper.

Pang F, Wang L-P, Jin Y, Guo L-P, Song L-P, Liu G-M, et al. Transcriptome analysis of *Paecilomyces hepiali* at different growth stages and culture additives to reveal putative genes in cordycepin biosynthesis. Genomics 2018; 110: 162–170.

Instead, our paper reports morphology and molecular mycology findings and addresses the multiple genotypes of *O. sinensis* and differentially cooccurring fungi in the stromata, SFPs densely covered with numerous ascocarps, and ascospores of natural *C. sinensis*. Clearly, our paper does not directly address pharmacological constituents and therapeutic functions, although cordycepin may be produced by cocolonized fungi and may contribute to the overall profile of the pharmacological functions of natural *C. sinensis*.

Reviewer #2 commented: “The abstract is too simple to set out the main opinion of this paper”

Authors’ response: We have followed the Reviewer’s comment and thoroughly revised the abstract to cover the results present in our manuscript. Please see the revised manuscript.

Reviewer’s comment: “Semi-ejected ascospore was considered to be one type that differ from the ejected ascospore. But be lack of the evidence in morphological and growth characteristics.”

Authors’ response: The lower-right panel of Fig 1 of our manuscript and Figure R5 of this response letter show numerous semiejected ascospores adhered to the surface of the asci. We stated in our manuscript (Lines 276‒279): “and the numerous semiejected ascospores that tightly adhered to the outer surface of asci (lower-right panel of Fig 1 after removing the upper layer of autoclaved weighing papers for collection of the fully ejected ascospores) were collected by gentle scraping using a disinfected inoculation shovel or ring [68].”

***Figure R5. The stromal fertile portion (SFP) with a mark “]” in red and the numerous semiejected ascospores that tightly adhered to the outer surface of asci (lower-right panel of Fig 1 of our manuscript).***

In addition to Figure R5, which presents the semiejected ascospores for perception by the naked eye, Fig 4B and 4E of our manuscript and Figure R6 of this response letter show the microscopic structures of semiejected ascospores. We have updated these 2 panels of Fig 4 in the revised manuscript by adding arrows in the figures to indicate the semiejected ascospores. We described them in our manuscript as follows (Lines 291‒298): “Panel 4B shows an optical microscopy image (10x) of several ascocarps, and Panel 4C shows a close-up optical microscopy image (40x) of an ascocarp. Ascocarps were stained with hematoxylin-eosin containing multiple ascospores, which revealed uneven staining of nuclei (dark blue–purple), consistent with the multicellular heterokaryosis of the *C. sinensis* ascospores reported by Bushley et al. [76]. Panels 4D and 4E provide confocal images close to the opening of the perithecia, showing ascospores gathering toward the opening of the perithecium (4D; bar, 50 μm) and a semiejected ascospore hanging out and adhered to the opening of the perithecium (4E; bar, 20 μm). Arrows in Panels 4B and 4E indicate semiejected ascospores.”

***Figure R6. Microscopic image of semiejected ascospores (Figs 4B and 4E of our manuscript).*** *Arrows indicate semiejected ascospores.*

Reviewer’s comment: “In instruction part, … the description and the definitions are confused, e.g. the stromal fertile portion (SFP), …”

Authors’ response: Figures 1 and 8 of our manuscript and Figure R7 of this response letter show the stromal fertile portion (SFP) of the *C. sinensis* stromata. We have updated Figs 1 and 8 by adding labels “]” in red and side-notes to indicate the SFPs in the figures.

***Figure R7. The stromal fertile portion (SFP) of natural C. sinensis is densely covered with numerous ascocarps (Figs 1 and 8 of our manuscript).***

Reviewer’s comments: “In instruction part, … Most sentences contain grammar mistakes. e.g. line 62-70, ‘ a dead Hepialidae moth larva’, should be the remains of Hepialidae moth larva, ‘intact intestine and head tissues, an intact, thick larval body cultivated insect-fungal complex’... The expressions are redundant and very difficult to read. There are many similar problems in this part.”

And “It is very noted that this manuscript needs careful editing by expertise in English editing in pay attention to the spelling, grammar, sentence structure. Otherwise, the content can’t be clearly present. Most sentences contains grammar mistakes in this ms.”

Authors’ response: Our manuscript has been edited 6 times by at least 3 editors of American Journal Experts (AJE) and 2 times by AI automated digital re-editing within the AJE system. We have been granted editing certificates (Figure R8). The revised manuscript was edited by 3 editors at the latest editing service.

In response to the comment “line 62-70, ‘a dead Hepialidae moth larva’, should be the remains of Hepialidae moth larva’”, we have followed the reviewer’s suggestion and revised the sentence in our manuscript (now Lines 78‒80).

In response to the comment ‘intact intestine and head tissues, an intact, thick larval body cultivated insect-fungal complex’, I think that the Reviewer is referring to Lines 80‒82 (*cf*. Figure R1 above). We have revised the sentence: “Studies have reported that the caterpillar body of *C. sinensis* contains an intact larval intestine, an intact and thick larval body wall with numerous bristles, head tissues, and fragments of other larval tissues [8‒12].”

***Figure R8. AJE editing certificates.*** *The AJE editing certificates were granted on May 6, 2021 (the upper panel), August 11, 2022 (the middle panel), and September 6, 2012 (the lower panel).*

The anatomic structures have often been observed during specimen sample handling in relevant studies. We have cited 5 papers to support the findings. Reference #12 (attached to this submission) includes the anatomic image of the caterpillar body of natural *C. sinensis* (*cf*. Figure R1 above in this response letter). However, that journal owns the copyright of this anatomic image. We may request reuse permission for the anatomic image (for a fee) or repeat the anatomy experiment if the reviewers believe that the anatomic image is critical and absolutely necessary for our audience to visualize the structure.

Reviewer’s comment: “In method part, the statement is too simple. Be deficient in descriptions of agents, materials, protocols, etc reagents, materials, protocol. And references are not fully cited. e. g. before the extraction, how the samples were stored? How to extract?”

Authors’ response: We have followed the reviewer’s comments and added the subsection “Reagents” to the Methods section. The detailed equipment information is described in the corresponding sections. We also checked the experimental protocols and added additional information that has been published previously in the cited papers regarding the experimental procedures, including sample cleaning, transportation, storage and genomic DNA extraction, in the corresponding sections.

Reviewer’s comment: “What gene was genotype primers designed with? Whether ITS1-5.8S-ITS2 segment is suitable for all the three types primers?”

Authors’ response: The genotyping primers were designed based on ITS1-5.8S-ITS2 sequences of GC- and AT-biased genotypes of *O. sinensis*. We designed more primers and tried them in different combinations in our experiments. We also tried the primers that other research groups used and published. In the preliminary experiments, we tested different pairing combinations of primers to test the amplification specificity and efficiency on multiple genotypic sequences and finalized the primer usage and pairing as described in our manuscript.

The GenBank database contains at least 668 ITS1-5.8S-ITS2 sequences of *O. sinensis*, most of which were obtained from uncultured natural *C. sinensis*. Among the sequences, some GC-biased Group-A sequences (Genotype #1) were obtained from pure cultures of *Hirsutella sinensis*, which have been morphologically identified, and their genomes have been sequenced. The variable sequences were used to design the genotype-specific primers.

Profs. David L. Hawksworth and Yi-Jian Yao, and their coauthors (Li et al. 2020b), stated: “the internal transcribed spacer (ITS) generally appears to evolve through concerted evolution and thus has been widely used in species identifications and phylogenetic studies of diverse organisms (e.g. Buckler and Holtsford 1996; Denman et al. 2000; Hřibová et al. 2011; Rouland-Lefevre et al. 2002).” Since 1999, ITS1-5.8S-ITS2 sequencing has been a molecular target in studying natural *C. sinensis* and *O. sinensis* fungus/fungi*,* and many variable ITS1-5.8S-ITS2 sequences of *O. sinensis* have been identified. In particular, Stensrud et al. (2007) and Zhang et al. (2013) studied numerous *H. sinensis* strains and reported genetic and phylogenetic findings based on the sequences of the ITS1-5.8S-ITS2 nrDNA and 5.8S gene. They confirmed that the transition mutants in Groups A‒C were correct sequences of *O. sinensis*.

To answer the Reviewer’s question, this section of our response letter includes some of the information that already appears in another section to keep our answer understandable as a whole story. Because a substantial portion of published papers are in Chinese, I am summarizing the useful information herein for your reference.

From 1999 through 2009, several studies examined ITS1-5.8S-ITS2 sequences in *C. sinensis* ITS sequences using universal primers and different *H. sinensis* strains as the study references (Zhao et al. 1999; Wang et al. 2000; Chen et al. 2001, 2002, 2004; Liu et al. 2001, Zhang et al. 2002; Wei et al. 2006; Hao et al. 2009; Yin et al. 2009). Based on the high homology between the *H. sinensis* ITS sequences and those obtained from natural *C. sinensis*, these papers concluded that *H. sinensis* is the sole, correct anamorph of *C. sinensis* that was later renamed *O. sinensis* in 2007.

Kinjo and Zang (2001) reported for the first time the detection of 3 phylogenetic clades of *C. sinensis* ITS variants in natural *C. sinensis* specimens collected from different production areas. They uploaded many ITS sequences of *C. sinensis* (*O. sinensis*) to GenBank.

Stensrud et al. (2005) disclosed a sequence AJ786590 (AT-biased Group-B) of *O. sinensis* and uploaded it to GenBank, which was discovered in natural *C. sinensis* by Engh (1999) in his or her unpublished graduate study at the University of Oslo. Stensrud et al. (2007) further analyzed the phylogeny of a total of 71 *C. sinensis* ITS1-5.8S-ITS2 sequences and 5.8S gene sequences, including the sequences discovered by Engh (1999) and Kinjo and Zang (2001), using a Bayesian algorithm and reported 3 phylogenetic clades of *C. sinensis* ITS sequences: Group A (*i.e.*, GC-biased Genotype #1) and Groups B‒C (*i.e.*, AT-biased Genotypes #4‒5). They stated that “Groups B and C (*i.e.*, AT-biased Genotypes #4‒5) … showed highly accelerated evolution of the 5.8S gene compared with group A (*i.e.*, GC-biased Genotype #1) … Such a large sequence variation of the 5.8S nrDNA far exceeds what is normally observed in fungi (and other organisms), even at higher taxonomic levels (genera and family)”. This was the first hypothesis that the variable ITS sequences belonged to independent fungi of phylogenetic variations “at higher taxonomic levels (genera and family)”.

Molecular pharmacologists Xiao et al. (2009) (corresponding with a senior member of the Chinese Mycological Association, Prof. Ping Zhu) used AT-biased genotype-specific primers and a nested PCR strategy and reported consistent occurrence of Group-A fungus (*i.e.*, Genotype #1) and differential occurrence of Groups-B‒C (*i.e.*, Genotypes #4‒5) of *O. sinensis* in the stroma and caterpillar body of natural *C. sinensis* specimens collected from different production areas. In addition, they identified the first sequence (EU555436) of AT-biased Genotype #6 and uploaded it to GenBank. Accordingly, they concluded that the variant sequences likely belonged to independent *O. sinensis* fungi.

Mycologists Zhang et al. (2009; corresponding with a former chairman of the Chinese Mycological Association) reported the detection of variable ITS sequences in natural *C. sinensis* specimens collected from Nyingchi of Tibet, compared with the *H. sinensis* sequences obtained from natural *C. sinensis* specimens collected from all other areas. They believed that the unique variable ITS sequence (GC-biased Genotype #3) represented an ancestor strain of *H. sinensis* and that Nyingchi of Tibet is the center of the evolutionary origin of the *O. sinensis* genus under the geography of *O. sinensis* evolution. Because Nyingchi in Tibet is geographically distant from the sample collection locations in Qinghai Province where we collected our samples, our study did not consider designing Genotype #3-specific primers.

Molecular biologists Zhu et al. (2010, 2012) reported the detection of a variable ITS sequence (GC-biased Genotype #2) cooccurring with Genotype #1 *H. sinensis* in the stromata of immature, maturing, and mature natural *C. sinensis* specimens collected from Kangding County of Sichuan Province. These 2 GC-biased genotypes showed distinct maturational patterns in the stromata during maturation of *C. sinensis* (*cf*. Fig 8 of our manuscript), suggesting that the sequences of the cooccurring GC-biased genotypes do not reside in a single fungal genome and indicating genomic independence of the GC-biased genotypes.

Zhu et al. (2012) summarized the findings of 6 mutant genotypes, GC-biased Genotypes #1‒3 and AT-biased Genotypes #4‒6, the sequences of which contain multiple scattered transition point mutations (*cf*. Figure R3 above).

Continuing on the phylogenetic study of *O. sinensis* by Stensrud et al. (2007), Chinese mycologists Zhang et al. (2013) analyzed 397 ITS sequences in another phylogenetic study and confirmed that the transition mutants in Groups A‒C were correct sequences of *O. sinensis*. However, Li et al. (2016) found that the Group-A clade reported by Zhang et al. (2013) contained several genetically variable GC-biased ITS sequences, having 81.6‒94.9% similarities to the ITS1-5.8S-ITS2 sequence of Genotype #1 AB067721 (*cf*. Table R1 above). Li et al. (2016) believed that Genotypes #7‒12 are genetically distinct from Genotypes #1‒6 that contain only multiple transition alleles (*cf*. Figure R3 above) and should be treated differently. Genotypes #7‒11 harbor multiple scattered transversion point mutations and relatively fewer insertion/deletion alleles, in addition to the transition alleles (Table R2 and Figure R4). The genotypic order of these variable ITS sequences was determined according to the allelic ratios of Transversion *vs*. Transition (Table R2). Genotype #12 harbors mainly multiple scattered insertion/deletion point mutations and much less transversion and transition alleles (*cf*. Table R2 and Figure R4). Thus, there are a total of 12 genotypes previously discovered in natural *C. sinensis*.

### Table R2 Mutant alleles in the ITS segments of *O. sinensis* Genotypes #7‒12.

| Genotype | GenBank Accession # | # of mutant alleles *vs*. AB067721 | | |  | Allelic ratio of | |
| --- | --- | --- | --- | --- | --- | --- | --- |
|  |  | In./De. | Transv. | Transit. |  | **Transv. *vs*. Transit.** | In./De. *vs.* Transv./Transit. |
| #7 | AJ488254 | 4 | 9 | 10 |  | **0.90** | 0.21 |
| #8 | GU246286 | 4 | 25 | 27 |  | **0.93** | 0.08 |
| #9 | GU246288 | 8 | 11 | 7 |  | **1.57** | 0.44 |
| #10 | GU246287 | 12 | 51 | 29 |  | **1.76** | 0.15 |
| #11 | JQ695935 | 29 | 75 | 40 |  | **1.88** | 0.25 |
| #12 | GU246296 | 17 | 8 | 5 |  | **1.60** | 1.31 |

*Note: [Modified from Table 3 of Li et al. (2016)]. “In./De.” refers to the sum of insertion/deletion mutations; “Transv.” And “Transit.” refer to transversion and transition mutations, respectively. The GC-biased Genotype #1 is represented by AB067721. Genotypes #7*‒*11 of the GC-biased transversion mutant O. sinensis are represented by AJ488254, GU246286, GU246288, GU246287 and JQ695935, respectively. Genotype #12 is represented by GU246296, the Insertion/Deletion mutant O. sinensis.*

GC-biased Genotype #7 (AJ488254) contains 9 transversion (Transv.) alleles and 4 insertion/deletion (Ins/Del.) alleles, in addition to 10 transition (Transit.) alleles, with an allelic ratios of 0.9 (Transv. *vs*. Transit.) and of 0.21 (Ins/Del. *vs*. Transv./Transit.) (Table R2). It was detected in the stroma of the natural *C. sinensis* specimen collected in Qinghai Province by Chen et al. (2011). Interestingly, they identified Genotype #1 (AJ488255) in the caterpillar body of the same *C. sinensis* specimen without detection of the coexistence of the 2 sequences in either of the compartments.

The sequences of Genotypes #8‒12 with the transversion and insertion/deletion point mutations (Tables R1 and R2) were directly uploaded to GenBank by Indian scientists with limited information. The sequences of Genotypes #8‒12 have not been detected in the samples collected from the China side of production areas (Gansu, Qinghai, Sichuan, Tibet and Yunnan provinces). Presumptively, Genotypes #8‒12 of *O. sinensis* may exist only in the specimens on the India or Nepal side of *C. sinensis* production areas, which are geographically far from where our study samples were collected in Qinghai Province. Thus, our study did not consider designing specific primers for GC-biased Genotypes #8‒12.

In addition to Genotypes #1‒12, our manuscript herein reports the discovery of additional genotypes, GC-biased Genotypes #13‒14, which feature large DNA segment substitutions and genetic material recombination between 2 parental fungi, and AT-biased Genotypes #15‒17, with multiple scattered transition alleles (*cf*. Tables 2‒3 and Figs 2 and 6 of our manuscript). We also found that the sequences of Genotypes #2‒17 do not reside in the genome assemblies ANOV00000000, JAAVMX000000000, LKHE00000000, LWBQ00000000 and NGJJ00000000 of Genotype #1 *H. sinensis* Strains Co18, IOZ07, 1229, ZJB12195 and CC1406-203, respectively, confirming the genomic independence of the *O. sinensis* genotypes and supporting the independent *O. sinensis* fungi hypothesis (Stensrud et al. 2007; Xiao et al. 2009).

In the results section of our manuscript, we described the pretest and formal study results for the genotype-specific primers (Lines 360‒382): “The universal primers *ITS5/ITS4* and the genotype-specific primer pairs *Hsprp1/Hsprp2*, *Hsprp1/Hsprp3*, or *Hsprp2/Hsprp3* were highly homologous to GC-biased Genotypes #1‒2 and/or #7. The primer design did not consider GC-biased Genotypes #3 and #8‒12 because the sequences were obtained from *C. sinensis* specimens collected from Nyingchi of Tibet and the Indian-Nepal side of the *C. sinensis* production areas, which are geographically distant from the sample collection locations in Qinghai Province used for our study. The genotype-specific primer pairs *HsATp1/ITS4*, *HsATp1/HsATp2*, and *HsATp1/HsATp3* were selected to amplify the AT-biased sequences of *O. sinensis* (*cf*. Table 1 and Fig 2). The amplification specificity and efficiency of the primer pairings have been previously tested repeatedly for the genotypes of *O. sinensis*. The PCR amplicons were subjected to cloning-based sequencing to explore the multiple mutant genotypes.

The primer pair *HsATp1/ITS4* is highly homologous to almost all AT-biased genotype sequences (100%/100%) but slightly less homologous to Genotype #5 (100%/95%) and AB067719-type sequences (95%/100%). The primer pair *HsATp1/HsATp2* is highly homologous to sequences of Genotypes #5, #15 and #17 (100%/100%) but less specific for Genotypes #4 and #16 (100%/80%). The primer pair *HsATp1/HsATp3* is highly homologous to Genotype #4 and #16 sequences (100%/100%) but less specific for Genotypes #5, #15 and #17 sequences (100%/85%). The homology of the primer *HsATp1* to the Genotype #6 sequence is unknown because all Genotype #6 sequences available in GenBank are short, probably due to the secondary structure/conformation within the ITS1 sequence close to its 5’ end and the ITS2 sequence close to its 3’ end. The primer *HsATp2* is 100% homologous to sequence EU555436 of Genotype #6, but *HsATp3* is less specific (85%) for EU555436. Thus, based on pretest and formal test results of the primer pairs in terms of the amplification specificity and efficiency, using all of these primer pairs in multiple PCR runs is essential for ensuring the successful amplification of all known genotypes of *O. sinensis* existing in the stroma, SFP (with ascocarps), and ascospores of *C. sinensis*.”

We noticed in our previous studies that some regions of ITS1, 5.8S gene and ITS2 sequences of the mutant genotypes may contain secondary structures (variable conformation), and not all primer pairs exhibit high amplification efficiency for all genotypes, even using some primers with high homologies based on the primary structure. Li et al. (2013) confirmed 3 different secondary structures of 5.8S genes of Groups A‒C (*i.e.*, Genotypes #1 and #4‒5) of *O. sinensis*. Thus, we decided to use more pairs of genotype-specific primers in our study to profile the cooccurring *O. sinensis* genotypes.

The answer to the Reviewer’s question is that the genotype-specific primers were designed based on ITS1-5.8S-ITS2 sequences, covering GC-biased Genotypes #1‒2 and #7 and AT-biased Genotypes #4‒6, prior to our formal study. On the other hand, we did not consider designing specific primers for GC-biased Genotypes #3 and #8‒12 because their sequences were identified particularly in the specimens collected from Nyingchi of Tibet (postulated as the center of *H. sinensis* evolutionary origin) or on the India or Nepal sides of the *C. sinensis* production areas, which are geographically distant from the locations of our sample collection in Qinghai Province, and because numerous studies did not report detection of the sequences of Genotypes #3 and #8‒12 in natural *C. sinensis* specimens collected from the major *C. sinensis* production areas in Sichuan and Qinghai provinces.

There has been a history of 20+ years of scientific discovery for multiple genotypes of *O. sinensis* since 1999, when the first ITS sequence was discovered. There is still a possibility that more variable genotypes could be discovered based on the findings from biochip-based SNP mass spectrometry genotyping studies (Zhu et al. 2010, 2012; Gao et al. 2011, 2012; Li et al. 2022). The genotype-specific primer design may need to be updated in the future according to the advances in *O. sinensis* studies.

Reviewer’s comment: “Why was touch-down PCR program used?”

Authors’ response: Touch-down PCR offers a simple and rapid means to optimize PCRs, increasing PCR amplification specificity without sacrificing amplification efficiency. High annealing temperatures in the initial PCR cycles increase the specificity of exponential amplification but may have low amplification efficiency. Decreasing the annealing temperature stepwise will gradually enhance the amplification efficiency.

Zhang et al. (2010a, 2010b) and Xia et al. (2015, 2016) conducted mycobiota and microbiota studies and demonstrated >90 fungal species spanning >37 fungal genera in natural *C. sinensis*. Many other studies detected 17 genotypes of *O. sinensis* in natural *C. sinensis* and showed high similarities among the variable ITS sequences. These findings have been validated by metagenomic and metatranscriptomic studies of natural *C. sinensis*. Thus, it is critical to our study to ensure high specificity of PCR amplification without sacrificing amplification efficiency. The solution is to use touch-down PCR.

Reviewer’s comment: “Line 120-121,‘the collected samples were cultivated in our laboratory (altitude 2,200 m) in Xining City, Qinghai Province of China, with the windows left open and under conditions with sufficient watering, sunshine and ventilation.’ The culturing conditions present unclear, including light, water, ventilation, soil.”

Authors’ response: The cultivation technique and conditions have been previously tested and published by the first author of the current study/manuscript: [68] Li Y-L. Preliminary study on the ascospores of *Cordyceps sinensis*. ***Edible Fungi Chin***. 2002;21(4): 9−10, in Chinese. The Qinghai Provincial government has published “The Local Standard Operation Procedure for the Laboratory Techniques of Collection, Purification, and Storage of the Ascospores of Natural *Cordyceps sinensis*” (ICS BeiAnHao: #249; DB63/-2007), officially released by the Qinghai Provincial Bureau of Quality and Technical Supervision on September 11, 2007.

Following the Reviewer’s comment, we have revised our manuscript (Lines 164−172): “Some of the mature *C. sinensis* specimens were harvested along with the outer mycelial cortices and soil surrounding the caterpillar body and replanted in paper cups in soil obtained from *C. sinensis* production areas (upper panel of Fig 1) and were cultivated in our laboratory (altitude 2,200 m) in Xining City, Qinghai Province of China [68‒69]. Because of the phototropism of natural *C. sinensis*, we kept the windows fully open, allowing sufficient sunshine and a natural plateau breeze blowing over the cultivated specimens in the paper cups. The room temperature was maintained naturally, fluctuating with the lowest temperature at 18−19°C during the night and the highest temperature at 22−23°C in the early afternoon. The humidity of our laboratory was maintained by spraying of water using an atomizer twice a day in the morning and evening.”

Reviewer’s comment: “The collection method of fully/semi-ejected ascospores present unclear.”

Authors’ response: We have added an image (lower-left panel of Fig 1 of our manuscript and Figure R9 below in this response letter) for the collection of fully ejected ascospores and revised the text describing the ascospore collection process. We described in Lines 173‒174: “Fully ejected ascospores of *C. sinensis* were collected using double layers of autoclaved weighing paper (lower-left panel of Fig 1).”

***Figure R9. Collection of fully ejected ascospores (lower-left panel of Fig 1 of our manuscript).***

The lower-right panel of Fig 1 of our manuscript and Figure R5 above in this response letter show the semiejected ascospores. We described in Lines 174‒178: “During massive ascospore ejection, numerous ascospores adhered to the outer surface of asci, as shown in the lower-right panel of Fig 1 after removing the upper layer of autoclaved weighing papers for collection of the fully ejected ascospores, and failed to be brushed away using an autoclaved brush; hence, these ascospores were instead gently scratched off using a disinfected inoculation shovel or ring and referred to as semiejected ascospores.”

Reviewer’s comment: “The part of result and discussion is redundant and too long, and lack a sense of reading and hierarchy. Suggested to thoroughly revise.”

Authors’ response: We have revised our manuscript and deleted some repeated statements.

A paragraph covering Lines 326‒329 in the manuscript submitted last time has been deleted.

Sentences within Lines 375‒382 in the manuscript submitted last time have been deleted and replaced with a short statement (Lines 530‒531 of the revised manuscript): “This may indicate that these GC-biased genotypes of *O. sinensis* may exist in the natural *C. sinensis* specimens in different production areas.”

Reviewer’s comment: “The references weren’t be completely correct and need to be formatted according to the requirement of Plos one.”

Authors’ response: We have rechecked the reference style and revised the list.
