# Supplementary material for: Differential coexistence of multiple genotypes of Ophiocordyceps sinensis in the stromata, ascocarps and ascospores of natural Cordyceps sinensis
Source: PLoS One. 2023 Mar 9;18(3):e0270776. doi: 10.1371/journal.pone.0270776 (PMC9997936; doi:10.1371/journal.pone.0270776)
Supplement: Attachment — Submitted filename: 2023-2-15 Responses to the reviewer #2.docx [file pone.0270776.s005.docx]

**Responses to the comments from reviewer #2**

Reviewer #2: In this manuscript, the authors concluded that multiple genotypes of *O. sinensis* coexisted differentially in the stromata, SFPs and 2 types of *C. sinensis* ascospores, along with *S. hepiali* and the AB067719-type fungus by molecular analyses. The results indicated the fungal components in different combinations and their dynamic alterations in the compartments of *C. sinensis* during maturation played symbiotic roles in the lifecycle of natural *C. sinensis*. This study would help to understand the development, the maturation of ascospores and artificial cultivation. However, some questions need be revised.

1. The bar in Figure 4A is not clear.

2. Modify the Bayesian tree in Figure 7. Some words are stacked together, and arrow marks are easy to misunderstanding.

3. Please unify the format of references, e.g. 74-76, 83 inconsistent with others.

Authors’ response:

1. The bar in Figure 4A is not clear.

We have modified Figure 4A with a thicker bar and an enlarged font.

2. Modify the Bayesian tree in Figure 7. Some words are stacked together, and arrow marks are easy to misunderstanding.

We have rearranged the arrows and marks in red and blue in Figure 7 to not stack the tree lines and bootstrapping values.

However, as we stated in the Methods section “**Phylogenetic analysis of ITS sequences**”, the Bayesian majority-rule consensus tree was inferred using MrBayes v3.2.7a software (the Markov chain Monte Carlo [MCMC] algorithm by Nanjing Genepioneer Biotechnologies Co. as a commercial technical service, because we have failed using our personal computers that were not able to perform such computational works. The tree lines and bootstrapping values in black in the tree were calculated using the company’s computer and software with a sampling frequency of 10^3^ iterations after discarding the first 25% of samples from a total of 1.1x10^8^ iterations. We are unfortunately not able to change the location of bootstrapping values in the Bayesian tree.

3. Please unify the format of references, e.g. 74-76, 83 inconsistent with others.

We have carefully checked the entire reference list and made corrections.
